# Detection of *Escherichia coli* O157:H7 and Shiga toxin 2a gene in pork, pig feces, and clean water at Jagalan slaughterhouse in Surakarta, Central Java Province, Indonesia

**DOI:** 10.14202/vetworld.2019.1584-1590

**Published:** 2019-10-19

**Authors:** Maria Kristiani Epi Goma, Alvita Indraswari, Aris Haryanto, Dyah Ayu Widiasih

**Affiliations:** 1 Graduate School of Veterinary Science, Faculty of Veterinary Medicine, Universitas Gadjah Mada, Yogyakarta, Indonesia; 2 Department of Biochemistry, Faculty of Veterinary Medicine, Universitas Gadjah Mada, Yogyakarta, Indonesia; 3 Department of Veterinary Public Health, Faculty of Veterinary Medicine, Universitas Gadjah Mada, Yogyakarta, Indonesia.

**Keywords:** *Escherichia coli* O157:H7, feces, pork, slaughterhouse, Shiga toxin 2a, water

## Abstract

**Background and Aim:**

The feasibility assessment of food products on the market becomes one of the milestones of food safety. The quality of food safety of animal origin especially pork need to get attention and more real action from the parties related and concerned. Since pork is also a source of transmission for the contagion of foodborne disease so that the study of the existence of several agents in the pork and its products become the benchmark of safety level. This study aimed to isolate, identify, and detect the Shiga toxin 2a (*stx2a*) gene from *Escherichia coli* O157:H7 in pork, pig feces, and clean water in the Jagalan slaughterhouse.

**Materials and Methods:**

A total of 70 samples consisting of 32 pork samples, 32 pig fecal samples, and 6 clean water samples were used to isolate and identify *E. coli* O157:H7 and the *stx2a* gene. Isolation and identification of *E. coli* O157:H7 were performed using culture on eosin methylene blue agar and Sorbitol-MacConkey agar media and confirmed molecularly with polymerase chain reaction to amplify the target genes *rfbE* (317 bp) and *fliC* (381 bp). The isolates, which were identified as *E. coli* O157:H7, were investigated for the *stx2a* gene (553 bp).

**Results:**

The results of this study show that of the total collected samples, *E. coli* O157:H7 was 28.6% in Jagalan slaughterhouse and consisted of 25% of pork samples, 31.25% of pig fecal samples, and 33.3% of clean water samples. The isolates that were identified to be *E. coli* O157:H7 mostly contained the *stx2a* gene, which was equal to 75%, and consisted of seven isolates from pork samples, seven isolates from fecal samples, and one isolate from clean water samples.

**Conclusion:**

*E. coli* O157:H7 was found in 28.6% of pork, pig feces, and clean water in Jagalan slaughterhouse and 75% of identified *E. coli* O157:H7 contained the *stx2a* gene.

## Introduction

*Escherichia coli* O157:H7 is a zoonotic bacterium and causes foodborne disease. The infection by these bacteria initially causes non-bleeding diarrhea accompanied by abdominal cramps. Then, it may develop into bloody diarrhea and hemolytic uremic syndrome (HUS), which causes kidney failure in humans [1]. At the beginning of 1993, there was an *E. coli* O157:H7 outbreak in Washington, Idaho, California, and Nevada, causing 56 people to suffer from HUS and 4 others to die [2]. In 1996, *E. coli* O157:H7 was found in Louisiana. The number of cases was 8-20 cases per year. In 2015, an outbreak caused 15 students to suffer from diarrhea and abdominal pain [3]. It was also reported that *E. coli* O157:H7 was found (15.8%) in the feces of patients who had kidney failure at RSUP Sanglah Bali, Indonesia [4]. Humans are infected by *E. coli* O157:H7 in various ways, one of which is by ingesting contaminated meat. Bacterial contamination of meat can occur from the process of cutting until it is being consumed. The meat can become contaminated in slaughterhouses from fecal contamination and other contaminations during the skinning process [5].

Pork is a foodstuff that is widely consumed by the people of Surakarta City, Central Java. It is provided by a large number of pigs slaughtered in Central Java’s slaughterhouse, the number of which continues to increase every year. The number of slaughtered pigs in this slaughterhouse in 2012 was 15,315, in 2013, it was 18,547, and in 2014, it was 18,443 [6]. Therefore, it is essential to conduct a research investigation on the detection of *E. coli* O157:H7 in pork, pig feces, and clean water at Jagalan slaughterhouse in Surakarta, Central Java, Indonesia. The results of this study may become a source of information for both veterinarians and the public, so pork can be processed, cooked, and consumed properly to prevent foodborne diseases. This research may also become the foundation or a reference for the government to improve the management of slaughterhouses and handling of pork in Jagalan slaughterhouse.

This study aimed to detect *E. coli* O157:H7 in pork, pig feces, and clean water in the Jagalan slaughterhouse and the Shiga toxin 2a (*stx2a*) gene from *E. coli* O157:H7 isolates isolated from pork, feces, and clean water from the Jagalan slaughterhouse.

## Materials and Methods

### Ethical approval

This study did not involve living creatures, either both animals and humans as research subjects, so it does not require ethical clearance approval.

### Sample collection

Sampling was performed at the Jagalan slaughterhouse Surakarta, Central Java Province, Indonesia. The total samples taken were 70 and consisted of 32 samples of pork, 32 samples of pig feces, and 6 samples of clean water from a water reservoir. Sampling was performed aseptically. Pork and pig feces were put into sterile plastic tubes while the water was put in a sterile bottle. The samples were taken to the Laboratory of Gadjah Mada University using a cool box for conventional testing of the culture on eosin methylene blue agar (EMBA) and Sorbitol-MacConkey agar (SMAC) media followed by molecular testing (polymerase chain reaction [PCR]).

### Isolation of *E. coli* O157

#### Meat, feces, and clear water sample

The bacterial isolation procedure in this study referred to Suardana *et al*. [4]. Ten grams of meat and fecal samples and 10 ml of the water sample were added to 90 ml of buffered peptone water (Conda CAT: 1403.00) 0.1%; then, the meat was mashed using a stomacher, while the feces and water samples were homogenized. Furthermore, as much as, 100 µl of diluted samples were spread on the surface of the EMBA media (Oxoid CM0069) using sterile bent glass, incubated at 37°C for 24 h. The growing colonies that showed a green metallic color with black spots in the middle counted as *E. coli* colonies and tested positive (+).

**Figure-1:**
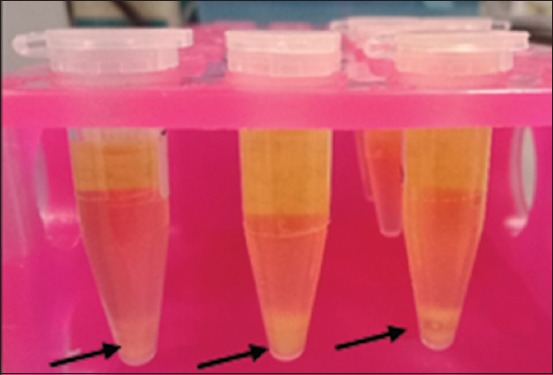
Results of isolates culture on brain heart infusion broth media, black arrows point at blurring occurrence.

Positive results on EMBA media from the three types of samples were cultured on SMAC selective media (Oxoid CM 0813) and were incubated at 37°C for 24 h. Colonies identified as *E. coli* O157 show characteristics of transparent colonies or colorless colonies, or non-sorbitol fermenting colonies. Colorless colonies were then cultured on brain heart infusion (BHI) media (Conda CAT: 1400.00) (800 µl), incubated at 37°C for 24 h and 200 µl of glycerol was added and stored at −20°C until needed for further examination. Positive bacteria growing on BHI media are characterized by turbidity in the media as shown in Figure-1. In this study, positive control of *E. coli* O157:H7 was used from the Inter-University Center Universitas Gadjah Mada.

### Isolation of bacterial genomic DNA

**Figure-2:**
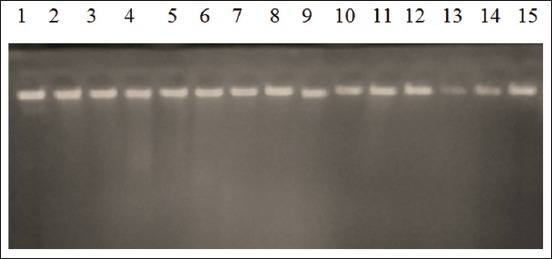
Results of bacterial DNA extraction from pig feces samples, line 1: F1, line 2: F3, line 3: F7, line 4: F8, line 5: F9, line 6: F10, line 7: F12, line 8: F13, line 9: F15, line 10: F20, line 11: F21, line 12-15: F29-F32.

Bacterial DNA isolation was done according to the Zymo Research supplier instructions. First, *E. coli* O157 isolates were harvested by centrifugation (12,000 rpm for 5 min), the supernatant was removed, and the cell pellets were mixed with 200 µl of the elution buffer DNA and vortexed. Then, the solution was mixed with 200 µl of biofluid and cell buffer and 20 µl of proteinase K. Following that, the mixture was vortexed and incubated in a water bath for 10 min at 55°C. Next, one volume (420 µl) of the Genomic Binding Buffer was added and vortexed for 10-15 s. Then, it was transferred into the Zymo-Spin IIC-XL Column in the collection tube, centrifuged at 12,000 rpm for 1 min, and the collection tube was discarded. After that, 400 µl of prewash buffer DNA was added into the spin column in a new collection tube, centrifuged at 12,000 rpm for 1 min; then, the liquid was removed (the collection tube was not removed). Next, 200 µl of gDNA wash buffer was added to the spin column and centrifuged at 12,000 rpm for 1 min. Then, the liquid and collection tube were removed. The spin column was transferred to the microcentrifugation tube, and 50 mL of elution buffer DNA was added. Following this, the solution was incubated for 5 min at room temperature and then centrifuged at maximum speed for 1 min to reach DNA elution. The spin column was then removed, and bacterial DNA was kept in the microcentrifugation tube. Electrophoresis was then performed and observed using a UV transilluminator to see the thickness of the extracted DNA, as shown in Figure-2. The pure DNA to be used immediately was stored at 4°C. Otherwise, it was stored in a −20°C freezer until needed [4].

### Identification of *E. coli* O157:H7

The target genes selected for *E. coli* O157:H7 identification were the *rfbE* and *fliC* genes. From the *rfbE* gene, the primers used were (F): 5’-TCTTTCCTCTGCGGTCCTA-3’ and (R): 5’-CAGGTGAAGGTGGAATGGT-3’ (product of PCR 317 bp). From the *fliC* gene , the primers used were (F): 5’-ATAATCTACGCCGCCAACT-3’ and (R): 5’-GACTCCATCCAGGACGAAA-3’ (product of PCR 381 bp) [7]. The *rfbE* gene encodes antigen O for serotype O157 and the *fliC* gene encodes antigen flagella H7 [8].

The PCR reaction for the detection of the *rfbE* and *fliC* genes was carried out at a total volume of 25 µl containing 12.5 µl Bioline mixture, 8.5 µl ddH_2_O, 2 µl template DNA, 1 µl primer (F), and 1 µl primer (R). Amplification was carried out on Thermal Cycler Model TC25/H machines with pre-denaturation conditions at 94°C for 5 min, followed by 40 cycles with denaturation reaction conditions at 94°C for 45 s, annealing at 56°C for 30 s, and polymerization at 72°C for 45 s. At the end, polymerization was added at 72°C for 10 min. After the amplification was complete, 5 µl of PCR product was mixed with 1 µl of loading dye and was electrophoresed on a 2% agarose gel filled with cyber safe dyes, along with a 100 bp DNA Ladder marker. Electrophoresis was performed at a voltage of 100 V for 55 min. The visualization of the band was done by employing a UV transilluminator and then using a digital camera to take a picture [4].

### Detection of stx2a gene

To detect the *stx2a* gene, we used primer (F): (5′-CGAGGGCTTGATGTCTATCAG.-3′) and (R): (5′-TCAGTATAACGGCCACAGTCC-3’) (product of PCR 553 bp), as previously conducted by Park *et al*. [9]. The PCR reaction was carried out on a total volume of 25 µl containing a 12.5 µl Bioline mixture, 8.5 µl ddH_2_O, 2 µl template DNA, 1 µl primary (F), and 1 µl primary (R). Amplification was performed on Thermal Cycler Model TC25/H machines with pre-denaturation conditions at 94°C for 5 min, followed by 40 cycles with denaturation reaction conditions at 94°C for 45 s, annealing at 56°C for 30 s, and polymerization at 72°C for 45 s. At the end, polymerization was added at 72°C for 10 min. After the amplification was complete, 5 µl of PCR product was mixed with 1 µl of loading dye and was electrophoresed on a 2% agarose gel filled with cyber safe dyes, along with a 100 bp DNA Ladder marker. Electrophoresis was carried out at a voltage of 100 V for 55 min. The visualization of the band was done by employing a UV transilluminator and then using a digital camera to take a picture [4].

### Statistical analysis

The data of the conventional and molecular bacterial isolation and identification, along with the observational results, were provided in the form of tables and figures. These results were then analyzed descriptively.

## Results and Discussion

*E. coli* O157:H7 is one of the strains of enterohemorrhagic *E. coli* that was first identified as a pathogenic bacterium in humans in 1992. This pathogen is associated with the occurrence of foodborne disease outbreaks, namely, hemorrhagic colitis (bloody diarrhea) [5]. Cows are animals that are considered the main reservoir of *E. coli* O157:H7 [2], but these bacteria are also found in other animals including sheep, goats, horses, dogs [10], and pigs [11].

**Figure-3:**
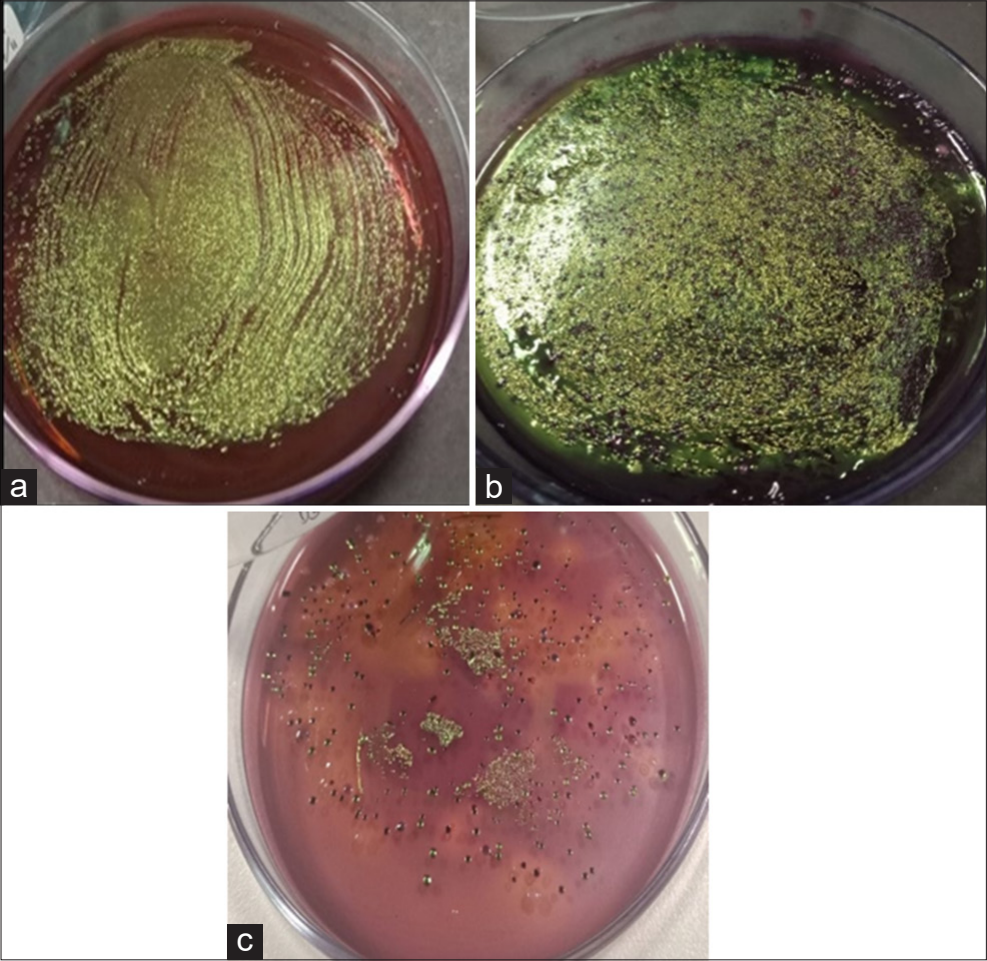
Results of sample culture on eosin methylene blue agar media; (a)feces sample with sample code F15; (b) pork sample from slaughterhouse with sample code D11; (c) clean water sample from Jagalan slaughterhouse reservoirs with AR2 code.

**Figure-4:**
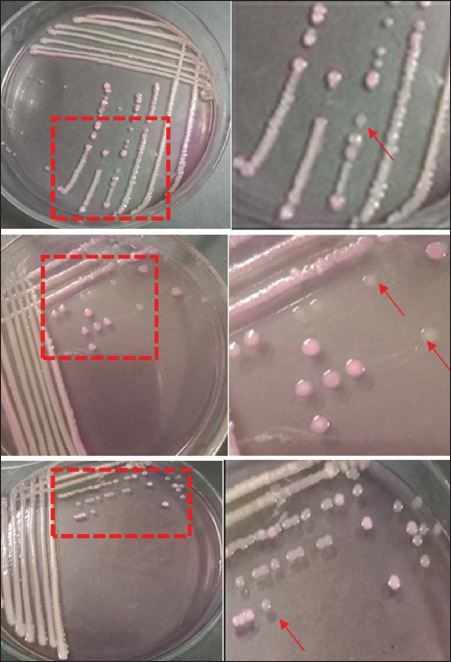
Results of bacteria isolation on Sorbitol-MacConkey agar media. The pictures from top to bottom followed by the enlargement of the colorless colony next to it in a row are pork sample; feces sample; water sample, and red arrows (↑) point at colorless colony.

**Figure-5:**
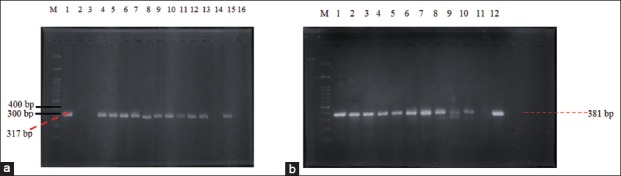
(a) Detection of gene *rfbE* (317bp) from pig feces on agarose 2%. M: Marker, line 1: Kontrol positif, line 2: F1, line 3: F3, line 4: F7, line 5: F8, line 6: F9, line 7: F10, line 8: F12, line 9: F13, line 10: F15, line 11: F20, line 12: F21, lines 13-16: F29-F32; (b): detection of gene *fliC* (381 bp) from pig feces sample on agarose 2%, M: Marker, line 1: positive control, line 2: F7, line 3: F8, line 4: F9, line 5: F10, line 6: F12, line 7: F13, line 8: F15, line 9: F20, line 10: F21, line 11: F29, line 12: F31.

**Figure-6:**
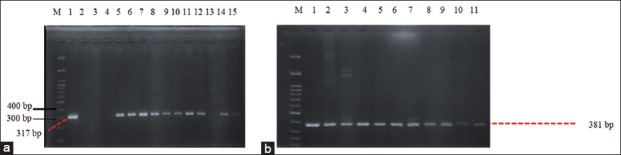
(a): Detection of gene *rfbE* (317 bp) from pork and water sample taken from Jagalan on agarose 2%, M: Marker, line 1: Positive control, line 2: D1, line 3: D3, line 4: D5, line 5: D7, line 6: D8, line 7: D9, line 8: D10, line 9: D11, line 10: D12, line 11: D15, line 12: D16, line 13: AR1, line 14: AR2, line 15: AL3; (b): Detection of gene *fliC* (381 bp) from pork and water sample taken from Jagalan on agarose 2%, M: Marker, line 1: Positive control, line 2: D7, line 3: D8, line 4: D9, line 5: D10, line 6: D11, line 7: D12, line 8: D15, line 9: D16, line 10: AR2, line 11: AL3.

The results of cultured samples showed that all samples (100%) were positive on EMBA media and consisted of 32 pork samples, 32 pig fecal samples, and 6 clean water samples. Positive results on EMBA media were characterized by the growth of green metallic sheen colonies (Figure-3). A total of 29 samples (11 pork samples, 15 fecal samples, and 3 clean water samples) were positive on SMAC media characterized by the growth of transparent colonies or colorless colonies (Figure-4) The confirmation results using the PCR method showed that there were 28.6% *E. coli* O157:H7 (20/70) in the slaughterhouse with 25% of pork samples, 31.25% of fecal samples, and 33.33% of clean water samples. The results were confirmed by the presence of the *rfbE* and *fliC* genes. Samples that positively contained the *rfbE* and *fliC* genes were characterized by the formation of bands at 317 bp and 381 bp, as shown in Table-1 and Figures-5 and 6.

**Table 1:** Conventional and molecular sample testing results.

Sampling location	Sample qty.	Sample type	EMBA (+)	SMAC (+)	*rfbE* gene (+)	*fliC* gene (+)	% *E. coli* O157:H7
Jagalan slaughterhouse	32	Feces	32	15	11	10	31.25
	6	Clean water	6	3	2	2	33.3
	32	Pork	32	11	8	8	25.0
Total			70	29	21	20	28.6

EMBA=Eosin methylene blue agar, SMAC=Sorbitol-MacConkey agar

The results of this study showed that *E. coli* O157:H7 was found in 31.25% of pig feces taken from Jagalan slaughterhouse, Surakarta, Central Java Province, Indonesia (Table-1). The results of this study were lower than the ones reported by Ateba and Mbewe [12], which stated that there was *E. coli* O157:H7 in 43.1% of pig feces (56/130) in the Northwest Province, South Africa. It was reported that the prevalence of *E. coli* O157:H7 in pigs in Chile was 10.8%, while in cattle, it was 2.9% [13]. Besides that, it was also reported that *E. coli* O157:H7 was found in 1.89% of cattle stool at a slaughterhouse in Ethiopia [14]. In Surakarta, Central Java, Indonesia, there have been no reports on the prevalence of *E. coli* O157:H7 in both pigs and cattle, but in Bali, Indonesia, it was reported by Suardana *et al*. [4] that the prevalence of *E. coli* O157:H7 in cattle feces was 5%.

The number of positive results found for *E. coli* O157:H7 in pig feces indicates that the infection is high in the pig population studied. The results of this study might be able to estimate the level of *E. coli* O157:H7 infection in a larger pig population in Surakarta, Central Java. However, it is quite challenging to retrace the origin of slaughtered pigs in the Jagalan slaughterhouse.

The results of this study also found *E. coli* O157:H7 in 25% of pork and 33.3% of clean water taken from the water reservoir in Jagalan, Surakarta (Central Java) (Table-1). The positive results of this study were higher than the results reported by Enabulele and Uraih [15]. They stated that there was *E. coli* O157:H7 in 6.94% of beef samples taken from a slaughterhouse in Benin City, Nigeria. However, it was also reported by Bouvet *et al*. [16] that the prevalence of verotoxigenic *E. coli* in pig carcasses in three slaughterhouses in France was higher than 80%.

The level of carcass and water contamination at Jagalan (slaughterhouse) in this study was quite high, at 25% and 33%, respectively. This result can also be correlated with the high positive results of *E. coli* O157:H7 found in 31.25% of pig feces. Similarly, Elder *et al*. [17] reported that *E. coli* O157:H7 was found in 17% of beef in the United States, which had a strong correlation between feces and meat contamination. The meats that were contaminated by *E. coli* O157:H7 in the slaughterhouses might be caused by various factors including fecal contamination on the meats, contaminated tools that were not sterilized anyhow, improper personal hygiene practices, and both air and rodent contamination [18].

Contamination by *E. coli* O157:H7 in water in slaughterhouses might be attributed to the condition of the reservoirs, which were not covered so that bacterial contamination could occur from pig feces. Contamination might also occur due to contact with the hands of butchers (workers) and anything else from the environment since the reservoir is open.

The pathogenicity of *E. coli* O157:H7 is associated with its ability to produce Shiga toxins, both Shigatoxin1 (*stx1*) and Shigatoxin2 (*stx2*). Bardasi *et al*. [19] stated that *E. coli* O157:H7 can produce both *stx1* and *stx2* or just one of the two toxins. The toxin prototype produced by *E. coli* O157:H7 has been found. Therefore, *stx1* is called *stx1a* and *stx2* is called *stx2a*. Two *stx1a* variants have been found, namely, *stx1c* and *stx1d*, while the *stx2a* variants that have been found are *stx2c*, *stx2d*, *stx2e*, *stx2f*, and *stx2g* [19].

Twenty isolates, which were identified with *E. coli* O157:H7, were followed by the detection of the *stx2a* gene. The results showed that 75% of *E. coli* O157:H7 isolates contained *stx2a*, with 87.5% isolates from pork samples, 70% isolates from fecal samples, and 50% isolates from clean water samples. Positive samples containing the *gene stx2a* are indicated by the formation of a band at the 553 bp position (Figure- 7), as shown in Table-2.

**Figure-7:**
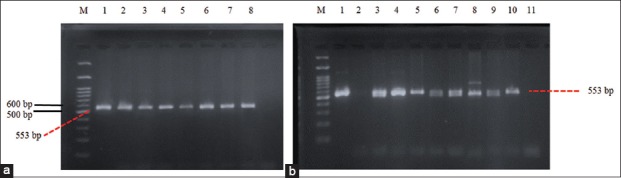
(a): Detection of gene *stx2a* from *Escherichia coli* O157:H7 isolates from pig feces sample on agarose 2%, M: Marker, line 1: Positive control, line 2: F7, line 3: F8, line 4: F9, line 5: F10, line 6: F12, line 7: F13, line 8: F15, line 9: F20, line 10: F21, line 11: F31; (b): Detection of stx2a gene from *E. coli* O157:H7 isolates from pork and clean water sample taken from Jagalan pada agarose 2%, M: Marker, line 1: Positive control, line 2: D7, line 3: D8, line 4: D9, line 5: D10, line 6: D11, line 7: D12, line 8: D15, line 9: D16, line 10: AR2, line 11: AL3.

**Table 2:** *st×2a* gene detection result.

Sampling location	Sample type	*E. coli* O157:H7	*st×2a* gene (+)	% *st×2a* gene
Jagalan slaughterhouse	Feces	10	7	70.0
	Clean water	2	1	50.0
	Pork	8	7	87.5
Total		20	15	75.0

*E. coli* O157:H7 isolates found in this study mostly contained gene *stx2a*, which was 75% of the sample (15/20) (Table-2). The results of this study are almost the same as those reported by Ateba and Mbewe [12], who said that the prevalence of *stx2* produced by *E. coli* O157:H7 isolates was 70.96% of pig fecal samples (22/31). Avery *et al*. [20] also reported that 79.2% of 24 *E. coli* O157:H7 isolates (19/24) isolated from beef carcasses produced *stx2*, 8.3% of the isolates (2/24) produced *stx1* and *stx2*, and the other 12.5% (3/24) did not produce *stx1* nor *stx2*. Then, 51.85% of *E. coli* isolates isolated from raw fish and fish that were ready to eat in Ludhiana, Punjab, produced *stx2 (*28/54) [21]. According to Melton-Celsa [22], *stx2a* is more toxic than *stx1a*. This toxicity difference is proved by endothelial cells, which, when exposed to HUS, are more sensitive to *stx2a* than *stx1a*. In addition, Frank *et al*. [23] also stated that the outbreak of HUS disease in Germany in 2011 was closely related to *stx2a*.

## Conclusion

Based on the results of isolation and identification, it can be concluded that there was a 28.6% contamination of *E. coli* O157:H7 found in Jagalan slaughterhouse, Surakarta city. The contamination was found in pig feces, pork, and water at 31.25%, 25%, and 33.3%, respectively. Of the 20 isolates of *E. coli* O157:H7 detected in the slaughterhouse, 75% of them contained gene *stx2a*.

## Authors’ Contributions

MKEG, AI, AH, and DAW designed the study. MKEG, AI, and DAW contributed in the field surveys, which consisted of taking and examining research samples conventionally, while AH contributed in the molecular sample testing (PCR). Allauthors wrote, edited, read, and approved the final manuscript.

## References

[1] Ferens W.A, Hovde C.J (2011). Escherichia coli O157:H7:Animal reservoir and sources of human infection. Foodborne Pathog. Dis.

[2] Segura A, Auffret P, Bibbal D, Bertoni M (2018). Factors involved in the persistence of a Shiga toxin-producing *Escherichia coli* O157 :H7 strain in bovine feces and gastrointestinal content. Front Microbiol.

[3] Louisiana Office of Public Health (2017). *E. coli* O157:H7*E. Coli* Annual Report.

[4] Suardana I.W, Artama W.T, Asmara W, Daryono B.S (2010). Identification of *Escherichia coli* O157:H7 and detection of Shiga like toxin 1 and 2 genes from animal feces beef, and human feces. J. Vet.

[5] Fernandez T.F (2008). *E coli* O157:H7. Vet. World.

[6] Statistics of Jawa Tengah Province (2017). Number of Livestock Slaughtered in Slaughterhouse and Out of Slaughterhouse Reported by Kind of Livestock in Jawa Tengah, 2012-2015.

[7] Suria M.S, Adlin A.A.K, Afendy M.A.T, Zamri I (2013). Multiplex Polymerase Chain Reaction (PCR) efficiency in the detection of pathogenic *Escherichia coli* O157:H7. Int. Food Res. J.

[8] Bai J, Shi X, Nagaraja T.G (2010). A Multiplex PCR procedure for the detection of six major virulence genes in *Escherichia coli* O157:H7. J. Microbiol. Methods.

[9] Park Y.S, Lee S.R, Kim Y.G (2006). Detection of *Escherichia coli* O157:H7*Salmonella* spp. Staphylococcus aureus and Listeria monocytogenes in Kimchi by multiplex polymerase chain reaction (mPCR). J. Microbiol.

[10] Spickler A.R (2016). Enterohemorrhagic *Escherichia coli* Infections.

[11] Tseng M, Fratamico P.M, Manning S.D, Funk J.A (2015). Shiga *toxin-producing Escherichia coli* in swine:The public health perspective. Anim. Health Res. Rev.

[12] Ateba C.N, Mbewe M (2011). Detection of *Escherichia coli* O157:H7 virulence genes in isolates from beef, pork, water, human and animal species in the Northwest province, South Africa:Public health implications. Res. Microbiol.

[13] Borie C, Monreal Z, Guerrero P, Sanchez M. L, Martinez J, Arellano C, Prado V (1997). Prevalence and characterization of enterohemorrhagic *Escherichia coli* isolated from bovines and healthy pigs slaughtered in Santiago Chile. Arch. Med. Vet.

[14] Abdissa R, Haile W, Fite A.T, Beyi A.F, Agga G.E, Edao B.M, Tadesse F, Korsa M.G, Beyene T, Beyene T.J, De Zutter L, Cox E, Goddeeris B.M (2017). Prevalence of *Escherichia coli* O157:H7 in beef cattle at slaughter and beef carcasses at retail shops in Ethiopia. BMC Infect. Dis.

[15] Enabulele S.A, Uraih N (2014). *Enterohaemorrhagic Escherichia coli*0157 :H7 prevalence in meat and vegetables sold in Benin city, Nigeria. AFR J. Microbiol. Res.

[16] Bouvet J, Bavai C, Rossel R, LeRoux A, Montel M.P, Ray-Gueniot S, Mazuy C, Arquillie're C, Vernozy-Rozand C (2001). Prevalence of verotoxin-producing *Escherichia coli* and *E. coli* O157:H7 in pig carcasses from three French slaughterhouses. nt. J. Food Microbiol.

[17] Elder R.O, Keen J.E, Siragusa G.R, Barkocy-Gallagher G.A, Koohmaraie M, Laegreid W.W (2000). Correlation of enterohemorrhagic *Escherichia coli* O157 prevalence in feces, hides, and carcasses of beef cattle during processing. Proc. Nat. Acad. Sci. USA.

[18] Laury-shaw A, Echeverry A, Brashears M.M (2009). Fate of *Escherichia coli* O157 :H7 in meat. J. Appl. Microbiol.

[19] Bardasi L, Taddei R, Fiocchi I, Pelliconi M.F, Toschi E (2017). Shiga toxin-producing *Escherichia coli* in slaughtered pigs and pork products. Ital. J. Food Safety.

[20] Avery S.M, Small A, Reid C.A, Buncic S (2002). Pulsed field gel electrophoresis characterization of Shiga toxin-producing *Escherichia coli*0157 from hides of cattle at slaughter. J. Food Prot.

[21] Gupta B, Ghatak S, Gill J.P.S (2013). Incidence and virulence properties of *E. coli* isolated from fresh fish and ready-to-eat fish products. Vet. World.

[22] Melton-Celsa A.R (2014). Shiga toxin (Stx) classification, structure, and function. Microbiol. Spectr.

[23] Frank C, Werber D, Cramer J.P, Askar M, Faber M, der Heiden M, Bernard H, Fruth A, Prager R, Spode A, Wadl M, Zoufaly A, Jordan S, Kemper M.J, Follin P, Muller L, King L.A, Rosner B, Buchholz U, Stark K, Krause G (2011). Epidemic profile of Shiga-toxin-producing *Escherichia coli* O104:H4 outbreak in Germany. N. Engl. J. Med.

